# Postvaccination Fever Response Rates in Children Derived Using the Fever Coach Mobile App: A Retrospective Observational Study

**DOI:** 10.2196/12223

**Published:** 2019-04-22

**Authors:** Sang Hyun Ahn, Jooho Zhiang, Hyery Kim, Seyun Chang, Jaewon Shin, Myeongchan Kim, Yura Lee, Jae-Ho Lee, Yu Rang Park

**Affiliations:** 1 Korea Human Resource Development Institute for Health and Welfare Cheongju Republic of Korea; 2 Yonsei University College of Medicine Seoul Republic of Korea; 3 Department of Pediatrics University of Ulsan College of Medicine Asan Medical Center Children’s Hospital Seoul Republic of Korea; 4 Mobile Doctor Co, Ltd Seoul Republic of Korea; 5 Department of Biomedical Informatics Asan Medical Center Seoul Republic of Korea; 6 Department of Emergency Medicine Asan Medical Center University of Ulsan College of Medicine Seoul Republic of Korea; 7 Department of Biomedical Systems Informatics Yonsei University College of Medicine Seoul Republic of Korea

**Keywords:** patient-generated health data, vaccination, postvaccination fever, digital health care, mobile app

## Abstract

**Background:**

Postvaccination fever is a mild adverse event that naturally improves without complications, but is highly prevalent and can be accompanied by febrile convulsions in some cases. These adverse effects may cause parents to delay or avoid vaccinating their children.

**Objective:**

This study aimed to identify postvaccination fever patterns and the ability of antipyretics to affect changes in these patterns from data collected from a mobile app named Fever Coach.

**Methods:**

Data provided by parents of feverish children derived from a mobile app, Fever Coach, were used to identify postvaccination fever patterns according to vaccinations and the use of antipyretic drugs. We selected single vaccination records that contained five or more body temperature readings performed within 48 hours of vaccination, and we analyzed postvaccination fever onset, offset, duration, and maximum body temperature. Through observing the postvaccination fever response to vaccination, we identified the effects of antipyretic drugs on postvaccination fever onset, offset, and duration times; the extent of fever; and the rate of decline. We also performed logistic regression analysis to determine demographic variables (age, weight, and sex) involved in relatively high fevers (body temperature ≥39°C).

**Results:**

The total number of Fever Coach users was 25,037, with 3834 users having entered single vaccination records, including 4448 vaccinations and 55,783 body temperature records. Most records were obtained from children receiving the following vaccinations: pneumococcus (n=2069); Japanese encephalitis (n=911); influenza (n=669); diphtheria, tetanus, and pertussis (n=403); and hepatitis A (n=252). According to the 4448 vaccination records, 3427 (77.05%) children had taken antipyretic drugs, and 3238 (89.15%) children took antibiotics at body temperatures above 38°C. The number of children taking antipyretics at a body temperature of 38°C was more than four times that of those taking antipyretics at 37.9°C (307 vs 67 cases). The number of instances in which this temperature threshold was reached was more than four times greater than the number when the temperature was 37.9°C. A comparative analysis of antipyretic and nonantipyretic cases showed there was no difference in onset time; however, offset and duration times were significantly shorter in nonantipyretic cases than in antipyretic cases (*P*<.001). In nonantipyretic cases, offset times and duration times were 9.9 and 10.1 hours shorter, respectively, than in antipyretic cases. Body temperatures also decreased faster in nonantipyretic cases. Influenza vaccine-associated fevers lasted relatively longer, whereas pneumococcus vaccine-associated fevers were relatively short-lived.

**Conclusions:**

These findings suggest that postvaccination fever has its own fever pattern, which is dependent on vaccine type and the presence of antipyretic drugs, and that postvaccination temperature monitoring may ease fever phobia and reduce the unnecessary use of antipyretics in medical care.

## Introduction

The World Health Organization and the Korean Centers for Disease Control and Prevention recommend at least 10 and 14 vaccines, respectively, for routine immunization of children [1]. Despite benefits in preventing serious infectious diseases, vaccinations are also associated with a risk of adverse events in patients. Fever is the most commonly reported adverse event [2,3]. The immune system’s lymphocyte and polymorphonuclear leukocyte functions improve at body temperatures between 38°C and 39°C. Therefore, the presence of postvaccination fever indicates that the immune system is functioning, but not due to a pathologic reaction [4-6]. There is no agreed definition of a postvaccination fever, but the gold standard definition of a fever is a rectal temperature of 38°C or above. Several studies report varying timeframes for postvaccination fever, ranging from within 32 hours, 48 hours, and 4 days of vaccination [7-9].

Postvaccination fever is a mild adverse event that is usually self-limiting over a few days without any specific complications. However, it is highly prevalent and, in some cases, accompanied with febrile convulsions. Furthermore, postvaccination fever may serve as an indicator of infectious disease. For these reasons, postvaccination fever can cause excessive anxiety in parents and caregivers [10-12]. Previous studies have indicated that parent perceptions and fear of fever have not significantly changed over the past 20 years and are still common in the Korean population [13-15]. Postvaccination fever phobia could lead to unnecessary testing, treatment (including the overuse of antipyretics), and emergency department visits, which increase both medical costs and the possibility of side effects [7,16].

Although fever can occur after every vaccination, a higher incidence of fever has been observed in patients receiving pneumococcal and DTaP (diphtheria, tetanus, and pertussis) vaccines than in those receiving other vaccines [17-19], indicating that fever patterns appear to be vaccine-specific. Many febrile diseases have specific fever patterns and progression (continuous, intermittent, remittent) that aid in understanding the pathophysiology of each disease and help to make diagnoses and therapeutic judgments [20,21]. Therefore, knowing the pattern and progress of postvaccination fever is likely to help address issues due to misperceptions regarding vaccination. There have been studies reporting on postvaccination fever frequency, but relatively few studies have reported postvaccination fever patterns [3,20-24]. However, many typical fever patterns can be changed through ingestion of antipyretics such as acetaminophen and steroidal anti-inflammatory drugs. The principal action of these drugs is inhibition of the enzyme cyclooxygenase and reduction of the levels of prostaglandin E_2_ within the hypothalamus [25]. However, data regarding postvaccination fever patterns and progress are difficult to obtain, with most postvaccination fever-related medical care occurring in outpatient settings where accurate body temperature recordings are not easily obtainable.

Patient-generated health data (PGHD) acquired through high-mobility mediums such as mobile phones, Internet of Things, wearable devices, and mobile apps have recently emerged as alternative methods for collecting data [26-31]. Some studies have shown that these PGHD have improved patient treatment, and studies are underway regarding how PGHD can be linked to routine treatment [32,33]. With immunization, however, obtaining longitudinal and continuous data is difficult. This study aimed to investigate the fever patterns of postvaccination fever through retrospectively analyzing PGHD obtained using Fever Coach, a fever management mobile app, and to analyze changes in fever patterns with the use of antipyretics.

## Methods

### Mobile App Description

Fever Coach is a mobile health care app developed by Mobile Doctor for parents with a feverish child. The app is based on pediatric thermal standards; it assesses a child’s condition based on user input and provides guidelines for antipyretic use. The app provides services that support parents’ effective and accurate control of common fever symptoms. Fever Coach provides several data services related to managing fever in children, such as microdust concentration status, body temperature information depending on geographical area, disease epidemic alerts, and pediatric health information. The app was made available as a free download from the Google Play Store and Apple App Store. As of June 31, 2017, 197,555 people had registered their child with the app.

**Figure 1:**
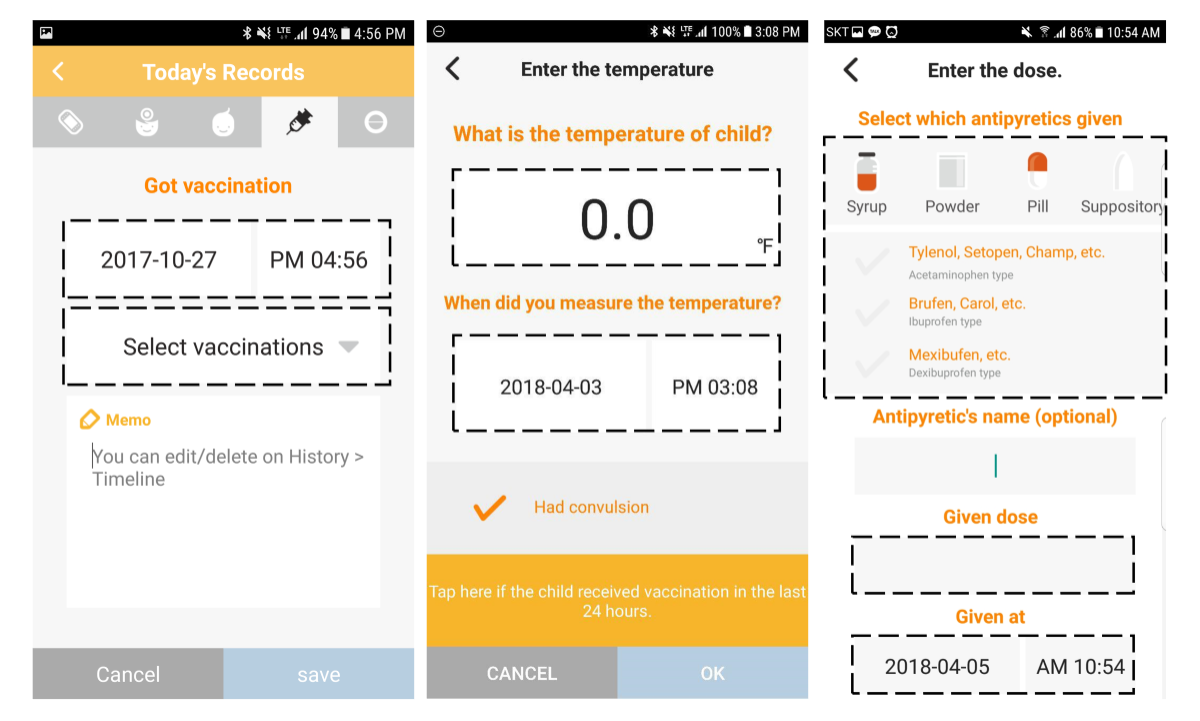
Screenshots of vaccination and fever response in the Fever Coach app. The left screen (“Today’s Records”), middle screen (“Enter the temperature”), and right screen (“Enter the dose”) have areas for user-input data. The functions corresponding to vaccination data, fever response, and antipyretic data in the three screens are indicated with dotted boxes. The original app showed Korean menu names; for international use, they have been translated into English.

We collected vaccination records and records of subsequent postvaccination fever responses and the antipyretics administered. Figure 1 shows the detail screens of the Fever Coach app and the types of data users can enter. All screens of Fever Coach are in Multimedia Appendix 1. The “Today’s Records” function provides information for vaccination records. The “Enter the Temperature” function allows the user to provide data concerning the fever response. The “Enter the Dose” function allows the user to provide records of antipyretic use.

### Study Design

To identify vaccination and antipyretic effects on postvaccination fever response patterns, we analyzed the logs of all users who signed up and registered their child between July 2015 and June 2017. Postvaccination fever usually lasts less than 48 hours [34]; therefore, we examined fever response and antipyretic administration data for 48 hours after the vaccination date entered by the user. For postvaccination fever response analyses, we defined the onset of fever as the point when the body temperature exceeded 38.0°C, and the offset as the point when the body temperature fell below 38.0°C [35,36].

**Figure 2:**
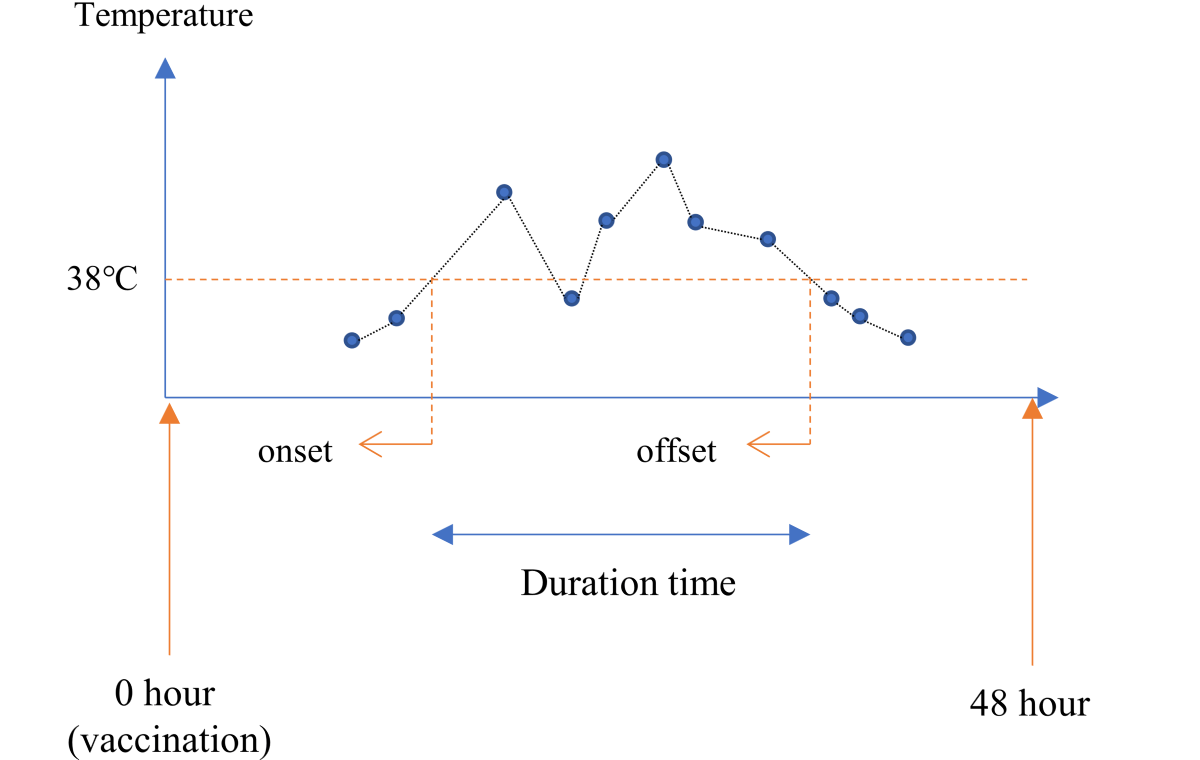
Onset, offset, and duration time definitions. The x-axis represents the time since vaccination and the y-axis represents the body temperature. The blue dot represents the actual body temperature. The black line between the blue dots is the imputated body temperature.

Onset time was defined as the time between vaccination and the point at which body temperature exceeded 38.0°C. If the first record was above 38.0°C, the time from vaccination to the registration of the data was defined as the onset time. Similarly, offset time was defined as the time between vaccination and the last point at which the body temperature fell below 38.0°C. The duration time was defined as the time elapsed between onset and offset points (Figure 2). Body temperature values were obtained using linear imputation techniques when they were missing between two actual body temperatures in the neighbors (Figure 2). We used this linear imputation technique on the assumption that the fever progression would show linear characteristics.

This study was approved by the Institutional Review Board of the Asan Medical Center (IRB no. 2018-0179; Seoul, South Korea). The need for informed consent was waived by the Ethics Committee because this study used routinely collected log data that were anonymously managed at all stages, including during data cleaning and statistical analyses.

### Data Collection and Analysis

**Figure 3:**
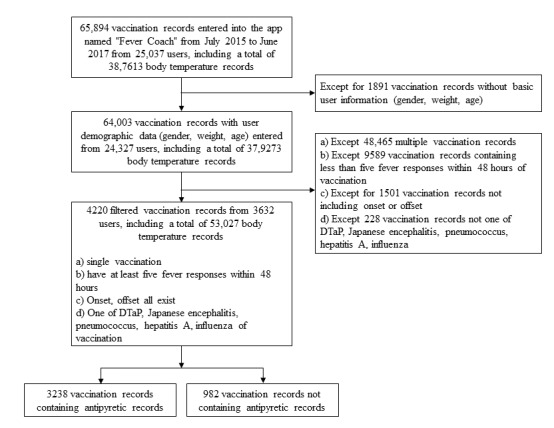
Data collection flowchart.

The app allows users to enter data regarding vaccinations, body temperatures, and antipyretic drug administration history. The vaccination record consists of the type and time of vaccine given to a particular child. The body temperature record consists of body temperature and measurement time for a specific child. The antipyretic drug administration record consists of antipyretic agent type, dose, and time. Figure 3 shows the target population selection flow of the study. From July 2015 to June 2017, 65,894 vaccination records were entered, of which 64,003 were vaccination records with basic user information (sex, weight, and age). Among them, the number of single vaccination records was 15,538. To obtain a sufficient number of body temperature recordings and significant onset and offset values, these data were filtered into 5949 cases with at least five body temperatures recorded within 48 hours after vaccination. Of these, 4448 comprised data-containing points corresponding to onset and offset times. Among these data, bacillus Calmette-Guérin, hepatitis B, measles-mumps-rubella (MMR), *Haemophilus* *influenzae* type B (Hib), polio, rotavirus, and chickenpox were excluded from the analysis because the number of vaccination records was less than 100; therefore, only data regarding DTaP, Japanese encephalitis, pneumococcus, hepatitis A, and influenza were analyzed. Although DTaP and MMR vaccines are combination vaccines, we considered these combination vaccines to be single vaccinations because they are single shots that can be distinguished from multiple vaccinations that involve multiple shots and that vaccines contain a variety of ingredients that can cause side effects [37].

The analysis was undertaken by grouping the data into 3238 cases with antipyretic records and 982 cases with no antipyretic records. For each vaccination record, the sex and age of the child were also collected.

To confirm the postvaccination fever differences between vaccination and antipyretic drug administration, we performed statistical analysis. To compare differences in onset times, offset times, duration times, and maximum temperatures, an independent sample *t* test was used to determine the degree of difference, while the *P* value for a two-sided test was used to test for significance. To observe the process of fever for each vaccine record, the onset time was defined as reference time 0, and body temperature values recorded over the previous 3 hours and the following 24 hours were obtained. Cases were grouped according to whether they were given antipyretic drugs and were reclassified according to vaccine type. Additionally, an ANOVA test was performed to compare maximum temperature and fever duration among the vaccine types. We used a Dunnett T3 post hoc test because the *P* value of the Levene test was less than .001, which indicated a violation of the assumption of homogeneity of variance. Lastly, binary logistic regression analysis was performed to determine demographic variables (age, weight, and sex) involved in relatively high fevers (body temperature ≥39°C). We performed multiple regression tests to identify multicollinearity between age and weight. If the variance inflation factor (VIF) was less than 10, it was considered that there was no multicollinearity for those variables. Data were processed and analyzed using R version 3.5.0, SPSS 21.0, and Python 3.6 (including packages of Pandas 0.22.0, NumPy 1.14.3, and Jupyter 1.0.0).

## Results

### Overall Characteristics

During the 24 months of the Fever Coach operation, a total of 25,037 users recorded 65,894 vaccinations involving 387,613 body temperature records for 25,608 children. Of the 64,003 vaccination records for which a child’s basic information was available, 15,538 (24.27%) were single vaccination records. Of the total number of enrolled children, there were 3834 (14.97%) children with five or more body temperature records at onset and offset, with 4448 vaccination records and 55,783 body temperature records (Table 1). The age at vaccination was significantly different in relation to the vaccine types. The proportion of males in this study was 60.12% (2193/3648). The proportion of vaccination records with antipyretic drugs was 77.05% (3427/4448). The majority of records were from children receiving the following vaccinations: pneumococcus (n=2069), Japanese encephalitis (n=911), influenza (n=669), DTaP (n=403), and hepatitis A (n=252). Each of the remaining single vaccinations had less than 100 records each.

**Table 1:** Basic characteristics according to vaccine type and the presence of antipyretic drugs.

Type of vaccination (number of records, number of children)	Age (months)		Sex (male), n (%)	Weight (kg)		Vaccination records with antipyretics, n (%)	Body temperature records, mean (SD)	Body temperature (°C)	
	Median (IQR^a^)	Mean (SD^b^)		Median (IQR)	Mean (SD)			Median (IQR)	Mean (SD)
BCG^c^ (16, 13)	6.6 (21.5)	13.1 (13.3)	8 (61.5)	7.7 (15.0)	8.6 (3.7)	10 (62.5)	10.3 (5.0)	37.7 (0.9)	37.6 (0.8)
Chickenpox (31, 28)	14.3 (6.2)	18.6 (12.3)	19 (67.9)	10.1 (35.0)	11.3 (4.7)	29 (93.5)	14.0 (7.7)	38.1 (1.0)	38.1 (0.8)
DTaP^d^ (394, 352)	13.3 (14.8)	14.9 (13.0)	212 (60.2)	9.5 (27.8)	9.5 (3.1)	318 (80.7)	12.7 (7.2)	37.9 (0.8)	37.9 (0.7)
Hepatitis B (61, 55)	10.7 (7.3)	14.5 (14.4)	37 (67.3)	9 (22.3)	9.7 (3.7)	54 (88.5)	12.1 (9.2)	37.9 (0.9)	37.9 (0.7)
Hepatitis A (247, 223)	19.8 (9.1)	20.4 (6.2)	130 (58.3)	11 (16.0)	11 (1.6)	229 (92.7)	14.3 (9.8)	38 (1.0)	38 (0.7)
Hib^e^ (47, 40)	15.3 (9.1)	14.4 (6.0)	20 (50.0)	9.7 (15.0)	9.4 (2.0)	43 (91.5)	12.1 (5.9)	37.9 (0.9)	37.9 (0.7)
Influenza (655, 587)	17.2 (7.9)	28 (20.8)	325 (55.4)	11.5 (39.0)	12.5 (4.3)	576 (87.9)	13.3 (8.1)	38 (0.9)	38 (0.8)
Japanese encephalitis (890, 793)	17.2 (7.9)	19.1 (8.1)	422 (53.2)	10.4 (23.5)	10.7 (1.8)	792 (89.0)	12.0 (7.5)	37.9 (0.8)	37.9 (0.7)
MMR^f^ (31, 28)	16.1 (10.0)	23.4 (15.8)	15 (53.6)	11 (19.0)	11.9 (2.8)	29 (93.5)	13.3 (6.2)	38.1 (1.1)	38.1 (0.8)
Pneumococcus (2034, 1771)	7.1 (11.0)	9.3 (8.1)	981 (55.5)	8 (32.0)	8.2 (2.5)	1323 (65.0)	12.3 (7.7)	37.7 (0.7)	37.8 (0.6)
Polio (19, 17)	12.3 (11.2)	12.1 (9.2)	11 (64.7)	8.8 (14.0)	9.3 (2.7)	11 (57.9)	11.5 (6.5)	37.7 (0.7)	37.7 (0.7)
Rotavirus (23, 22)	5.2 (4.4)	5.5 (3.1)	13 (59.1)	7 (11.5)	7.1 (1.7)	13 (56.5)	9.6 (5.1)	37.7 (0.9)	37.7 (0.5)
Total (4448, 3648)	13.5 (13.0)	15.4 (13.3)	2193 (60.1)	9.7 (3.5)	9.7 (3.2)	3427 (77.0)	12.5 (7.8)	37.7 (0.7)	37.9 (0.7)

^a^IQR: interquartile range.

^b^SD: standard deviation.

^c^BCG: bacille Calmette-Guérin.

^d^DTaP: diphtheria, tetanus, pertussis.

^e^Hib: *Haemophilus* *influenzae* type b.

^f^MMR: measles, mumps, and rubella.

### Antipyretic Drug Administration Pattern

**Figure 4:**
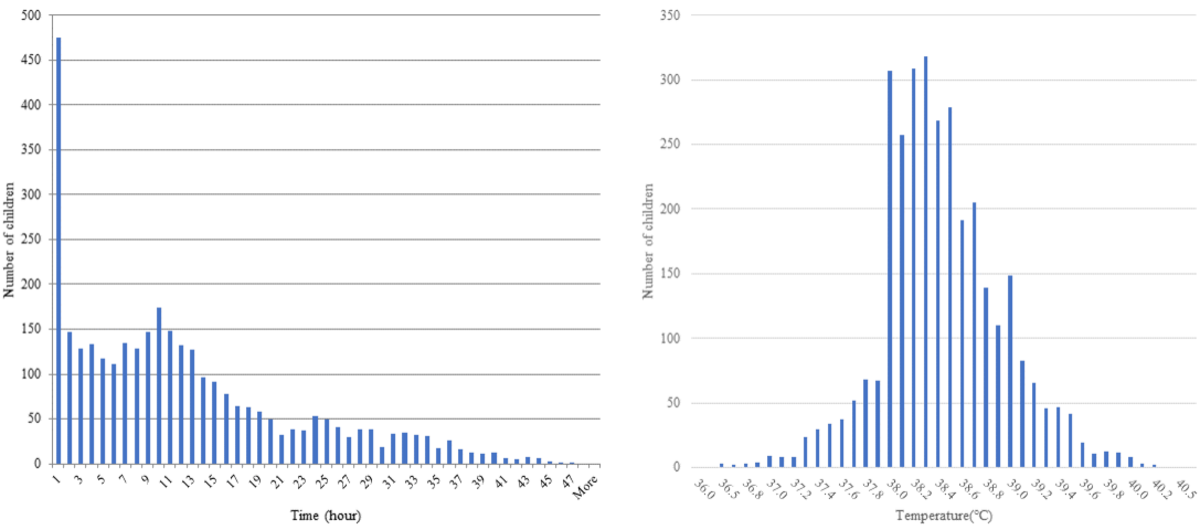
Time of first administration of antipyretic after vaccination and body temperature at the time of antipyretic use.

After vaccination, 14.66% (475/3238) of children were administered the first antipyretic drug within 1 hour, and more than 50% of children were administered antipyretic drugs within 10 hours. By 10 hours, the number of children treated with antipyretic drugs increased gradually, but decreased from 11 hours to less than 10% at 41 hours. A total of 2887 (89.16%) children used antipyretics when the postvaccination body temperature was 38.0°C or above (Figure 4). The number of children taking antipyretics at a body temperature of 38°C was more than four times that of those taking antipyretics at 37.9°C (307 vs 67 cases). The percentages of children who received antipyretic drugs were 0.71% (23/3238) and 0.49% (16/3238) at body temperatures lower than 37°C and above 40°C, respectively.

### Comparison of Onset, Offset, and Duration Times Among Vaccination Records With and Without Antipyretic Administration

**Figure 5:**
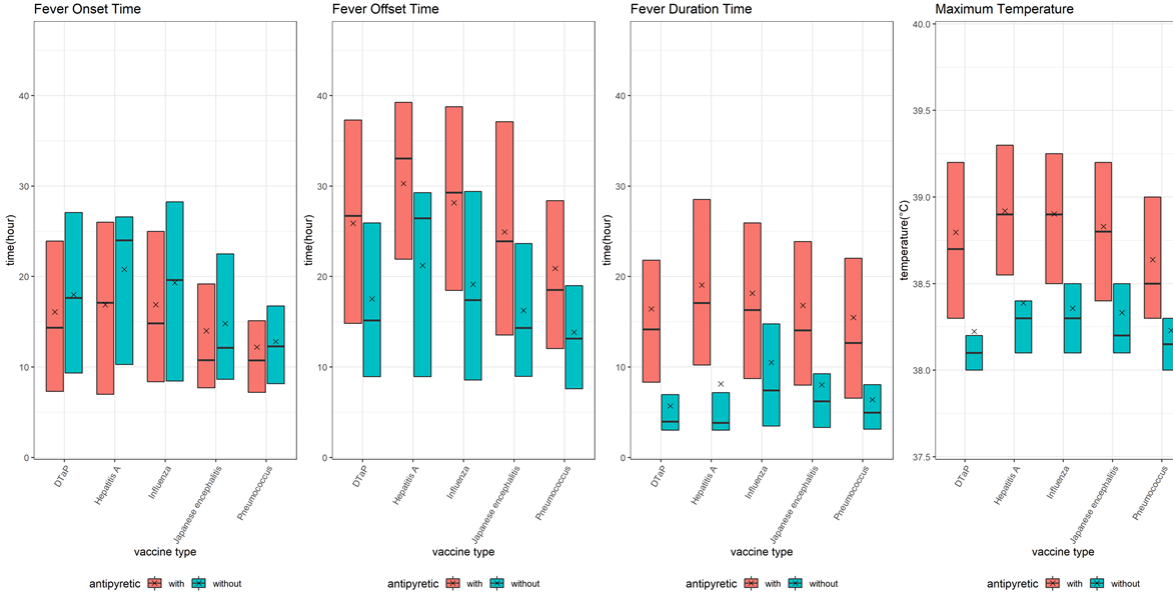
Comparison of onset, offset, duration times, and maximum temperatures among vaccine types, and the effects of antipyretics on postvaccination fever response. From left to right: box plots of onset, offset, duration times, and maximum body temperature are depicted. The bar indicates the median; x indicates the mean.

There were significant differences in offset times, duration times, and maximum temperatures between groups that had taken antipyretics and those that had not, but there was no significant difference in onset times (Figure 5). Differences between groups were marked, especially maximum temperatures that affected offset time and duration. Children vaccinated against hepatitis A showed the greatest difference in maximum temperature (mean 39.0ºC, SD 0.6ºC vs mean 38.4ºC, SD 0.4ºC in children who had been administered antipyretic drugs compared to children who had not been administered antipyretic drugs). The postvaccination fever duration was three times longer in children who were administered antipyretic drugs than in children who were not administered antipyretic drugs (mean 17.4, SD 11.8 hours vs mean 4.9, SD 7.5 hours, with antipyretic drugs compared to without antipyretic drugs).

Postvaccination fever differences between onset, offset, and duration times associated with each vaccine showed statistically significant differences in offset times and duration between groups (*P*<.001) (Multimedia Appendix 2). Offset and duration times for children who were not administered antipyretics were significantly shorter than for those who were administered antipyretics. There was no significant difference in onset time between the groups.

We found statistically significant differences in maximum temperature and fever duration among vaccines (both *P*<.001). In the postanalysis grouping, maximum temperature and fever duration decreased in the following order: hepatitis A, influenza, Japanese encephalitis, DTaP, and pneumococcus (Table 2).

We used logistic regression to determine the demographic variables involved in relatively high fevers (Table 3). There was no multicollinearity between age and weight (age VIF=4.38, weight VIF=4.38). Sex was not a significant variable in all groups, and age was not significant except in children who were administered the pneumococcus vaccine. Weight was statistically significant in all cases except in those receiving the hepatitis A vaccine.

**Table 2:** Comparison of maximum temperature and fever duration among vaccine types.

Vaccine type		Mean (SD)	*F*_4,4215_	*P* value^a^
**Maximum temperature (°C)**			94.7	<.001
	Pneumococcus	38.5 (0.5)		
	DTaP	38.7 (0.6)		
	Hepatitis A	39.0 (0.6)		
	Influenza	38.9 (0.6)		
	Japanese encephalitis	38.8 (0.5)		
**Fever duration (hours)**			62.8	<.001
	Pneumococcus	8.5 (10.5)		
	DTaP	11.3 (11.1)		
	Hepatitis A	16.4 (12.0)		
	Influenza	14.6 (12.3)		
	Japanese encephalitis	12.8 (11.7)		

^a^As a result of the post hoc test (Dunnett T3), maximum temperature and fever duration showed the following: Hepatitis A, influenza > Japanese encephalitis, DTaP > pneumococcus.

**Table 3:** Variables involved in relatively high postvaccination fever (≥39°C).^a^

Vaccine type and variable		B (SE)	*P* value	Adjusted odds ratio (95% CI)
**Pneumococcus**				
	Age	0.056 (0.014)	<.001	1.057 (1.029-1.086)
	Weight	0.210 (0.042)	<.001	1.233 (1.135-1.340)
	Sex	–0.096 (0.121)	.42	0.908 (0.716-1.152)
**DTaP**				
	Age	–0.004 (0.018)	.82	0.996 (0.960-1.033)
	Weight	0.234 (0.083)	.005	1.263 (1.075-1.485)
	Sex	–0.331 (0.231)	.15	0.718 (0.457-1.129)
**Japanese encephalitis**				
	Age	0.006 (0.012)	.59	1.006 (0.984-1.029)
	Weight	0.108 (0.052)	.03	1.114 (1.005-1.234)
	Sex	–0.070 (0.142)	.62	0.933 (0.706-1.233)
**Hepatitis A**				
	Age	0.036 (0.024)	.13	1.037 (0.989-1.088)
	Weight	0.011 (0.094)	.90	1.011 (0.841-1.217)
	Sex	0.158 (0.264)	.55	1.171 (0.698-1.966)
**Influenza**				
	Age	–0.016 (0.010)	.09	0.984 (0.966-1.003)
	Weight	0.104 (0.047)	.02	1.109 (1.102-1.216)
	Sex	0.002 (0.160)	.98	1.002 (0.732-1.372)
**All**				
	Age	–0.004 (0.006)	.43	0.996 (0.985-1.007)
	Weight	0.226 (0.025)	<.001	1.254 (1.195-1.316)
	Sex	–0.106 (0.071)	.13	0.899 (0.782-1.034)

^a^CI: confidence interval; DTaP: diphtheria, tetanus, pertussis; SE: standard error.

**Figure 6:**
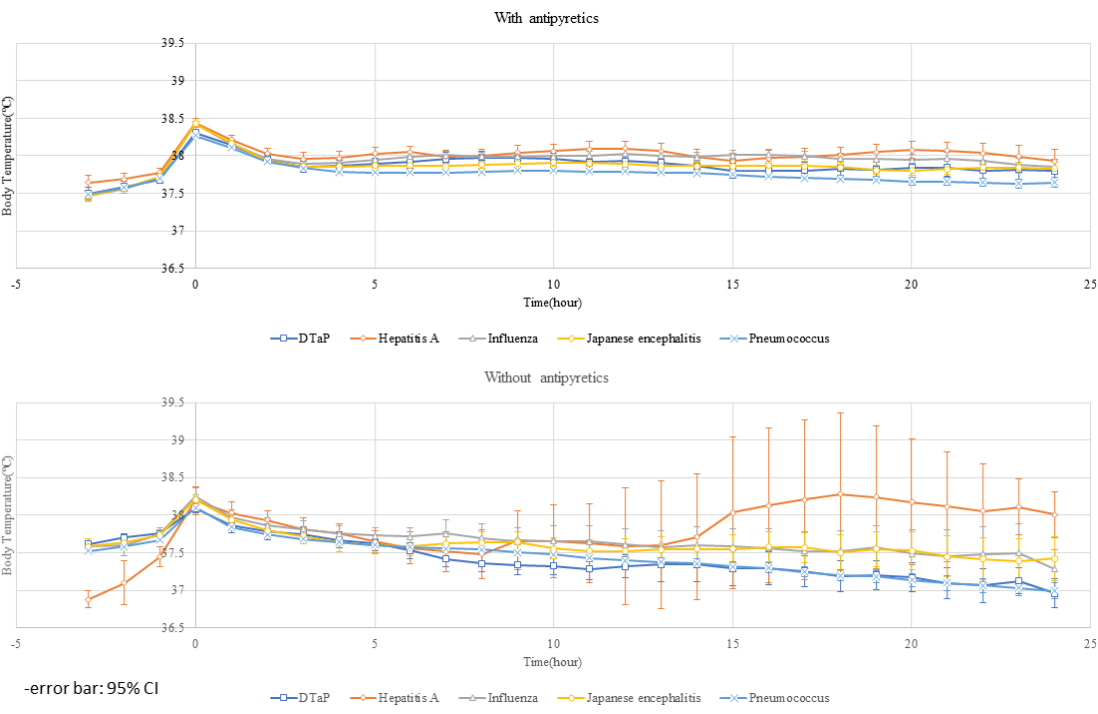
Body temperature graph over time for each vaccine showing the effects of antipyretic administration. The empty circle indicates the mean; the error bar represents a 95% confidence interval.

### Comparison of Fever Response

Figure 6 shows the result of plotting body temperature over time for each vaccine type and the effects of antipyretic drug administration. In cases where antipyretics were not administered, especially concerning DTaP and pneumococcus, the slope of the graph was steeper than in cases that included antipyretics, indicating that body temperature dropped more rapidly in children who were not administered antipyretic drugs (DTaP *R*^2^: .84 vs .00; pneumococcus *R*^2^: .83 vs .14). In the antipyretic use group, the mean body temperature was 37.5°C or above even after 24 hours, but in the nonantipyretic use group, the mean body temperature dropped below 37.5°C before 20 hours for all vaccines except hepatitis A. In comparing vaccines, we found that postvaccination fever associated with the influenza vaccine tended to be relatively long-lasting, and that the pneumococcus vaccine showed a relatively rapid decline.

### Comparison of the Effects of Antipyretics on Postvaccination Fever Response

Multimedia Appendix 2 shows the comparison of onset, offset, duration times, maximum temperatures among vaccine types, and the effects of antipyretics on postvaccination fever response. Postvaccination fever in children who were administered antipyretics exhibited a mean onset time of between 9.0 and 13.7 hours, a mean offset time of between 20.4 and 31.1 hours, a mean duration time of between 11.5 and 17.4 hours, and a maximum temperature of between 38.7°C and 39°C. The mean postvaccination fever onset time was not significantly different between children who were administered antipyretics and those who were not (mean 10.9, SD hours 9.9 vs mean 10.4, SD hours, 9.3 *P*=.12), but the mean offset time and duration were significantly different (offset time: mean 24.3, SD hours 13.4 vs mean 13.6, SD 9.9 hours, *P*<.001; duration: mean 13.4, SD 11.9 hours vs mean 3.2, SD 5.0 hours, *P*<.001). The maximum body temperatures at onset time were mean 38.8°C (SD 0.6°C) in the antipyretic group and mean 38.3°C (SD 0.5°C) in the nonantipyretic group, with a statistically significant difference between the two groups (*P*<.001).

## Discussion

### Principal Findings

We identified different postvaccination fever responses for each vaccine using data collected through the mobile app Fever Coach. For example, in relation to the hepatitis A vaccine, the postvaccination fever maximum temperature was statistically significantly higher and was of a longer duration than that for other vaccines (*P*<.001). We were also able to clearly identify differences among postvaccination fevers with and without antipyretic administration; offset, duration times, and maximum temperature were significantly different between groups (*P*<.001). Additionally, we presented new evidence from large-scale PGHD concerning fever duration and maximum temperatures reached after a single vaccination. The postvaccination fever duration was generally 48 hours but, for all single vaccinations, the longest postvaccination fever duration was mean 16.4 (SD 12.0) hours, and the highest maximum temperature was mean 39.0°C (SD 0.6°C). In terms of informatics, an additional contribution of this study is that the majority of users verified the pediatric fever level based on the PGHD. This study is original compared with previous studies for the following reasons: we used actual postvaccination fever response data without any clinical intervention; we analyzed large-scale postvaccination fever responses based on real-world data rather than relying on data from a small, clinical-based group; and our study used data derived from anyone using a mobile phone and not just from a specific hospital or specific patient area, which allowed us to analyze individual data covering a larger area than from data from a single institution or specific area.

### Characteristics of Postvaccination Fever and Its Natural Course According to Vaccine Type

Because most previous studies have been conducted to determine vaccine safety, they primarily discussed the frequency and severity of postvaccination fever and did not provide medical direction to parents or caregivers. This study showed that postvaccination fever associated with each vaccine has a unique fever pattern in terms of maximum temperature and duration.

Among all vaccines included in the study, the postvaccination fever in the nonantipyretic use group showed a mean onset time of 9.4 hours (pneumococcal vaccine) to 15.3 hours (hepatitis A vaccine), a mean offset time of 12.5 hours (pneumococcal vaccine) to 20.1 hours (hepatitis A vaccine), and a mean duration time of 2.5 hours (DTaP vaccine) to 6.1 hours (influenza vaccine) (details in Multimedia Appendix 2). These values are considered consistent with associated fever and vaccination administration if the fever occurs within 24 hours of vaccination and if the temperature falls within 24 hours after onset in general practice [38]. The characteristics of postvaccination fever according to vaccine type in all data were as follows: the maximum temperature was highest for children vaccinated against hepatitis A and lowest for children receiving the pneumococcal vaccine. Children receiving the pneumococcal vaccine are known to have a high incidence of postvaccination fever; therefore, it is not uncommon for clinical practitioners to give a warning before and after vaccination and recommend that parents administer antipyretic agents when fever occurs [19,39]. However, the results of this study showed that the mean temperature following pneumococcal vaccination was significantly lower than for other vaccinations, regardless of the use of antipyretics. Fever duration was longest for hepatitis A and influenza, followed by Japanese encephalitis, pneumococcus, and DTaP vaccine. Postvaccination fever associated with pneumococcal vaccine had a lower maximum temperature and a shorter duration than postvaccination fever associated with hepatitis A vaccine but fell below the fever onset temperature (38°C) within 24 hours after fever occurred. If parents were more aware of this natural course of postvaccination fever, they may be less anxious and reduce the use of antipyretics, thereby reducing unnecessary medical care [40,41]. We would expect parents to have a more positive outlook regarding vaccinations once their fears concerning fever had been more fully addressed [15].

Our results showed that, as a child’s weight increased, there was a high probability that a relatively high fever with a body temperature of less than 39°C would occur, regardless of age or sex (Table 3). It is known that the incidence of fever (especially a high fever with a body temperature of 39°C) tends to increase in children receiving the pneumococcal booster vaccination after 1 year of age, which is thought to be due to the booster effect, as the immune function matures and the number of vaccinations increase with age [24]. Here, however, only weight was significantly associated with the risk of high fever. Generally, a child’s age and weight are linearly correlated. The immune response to vaccination is known to be higher for girls than for boys [42,43], but there was no difference in the frequency of relatively high fevers between the sexes.

### Postvaccination Fever and Antipyretics

The duration of postvaccination fever in children who were administered antipyretics tended to be more prolonged than for those who were not administered antipyretics in this study. However, the actual body temperature at onset time and the maximum temperature were significantly higher in children who were administered antipyretics than in those who were not administered antipyretics (mean 38.8, SD 0.6 vs mean 38.3, SD 0.5, respectively, *P*<.001). Therefore, it is possible that if the period of decrease to the fever onset temperature (38°C) is prolonged, antipyretics could be responsible for inhibiting the immune response and extending the duration. It appears that the use of antipyretic drugs influences the offset time rather than the onset time, shortening the duration to offset time. Antipyretics were associated with a trend toward prolonged duration of illness in a group infected with *Shigella* *sonnei* and the influenza virus [44,45]. A systematic review indicated that the use of antipyretics in cases of malaria or viral diseases could shorten the duration of fever without prolonging the course of disease [38,39]. As noted, the effect of antipyretics on the course of postvaccination fever remains inconclusive, and the effect of antipyretics on postvaccination fever remains unknown. Based on the results of this study, it is possible that antipyretic use may prolong fever duration in children with postvaccination fever.

According to surveys on the use of antipyretics for postvaccination fever prevention and management by parents and caregivers, 11% of parents administered antipyretics for prophylaxis, and 64% of parents administered antipyretics for prevention or management within 48 hours of vaccination [46]. Here, after vaccination, 14.66% of children were administered their first antipyretic drugs within 1 hour and 16.2% of children were administered antipyretics for fever with a body temperature of less than 38°C (Figure 4). This value was higher than in previous studies and may be due to (1) differences in data used in the analysis, (2) cultural differences in the perception of fever, and (3) relative underestimation due to recall bias arising from retrospective survey methods used in prior studies. Also, many parents and caregivers were administering antipyretics for management of postvaccination fever when body temperatures reached 38°C, most likely because the app instructions advised parents not to administer antipyretics below a body temperature of 38°C but rather to administer antipyretics when the body temperature was above 38°C depending on the child’s condition. Various studies have been conducted on the risks and benefits of using antipyretics for postvaccination fever. Febrile convulsions are the most worrying situation for parents and caregivers when fever is present. To prevent convulsions, prophylactic antipyretics and routine antipyretics have been used during fevers, but with no effect on reducing febrile convulsions confirmed [23,39,47]. Additionally, several studies have suggested that antipyretics may be associated with decreased immunogenicity, and routine antipyretic administration is no longer recommended for postvaccination fever in countries such as Canada and New Zealand [22,24,48-51]. Alternatively, a systematic review has shown the difficulty of concluding that the reduction in immunogenicity due to antipyretic use does not fall below the seroprotective level and, thus, does not represent actual vaccine failure [48]. A high proportion of antipyretic use for postvaccination fever is believed to be due to fever phobia and a parent’s expectation that administering antipyretic drugs will ease a child’s discomfort rather than being based on persuasive scientific evidence [52]. Although antipyretic drugs had a temporary effect in lowering body temperature for a mean of 4.4 hours, it is not known whether their use reduced postvaccination fever-related discomfort. To guide the use of antipyretics for alleviating postvaccination fever-related discomfort based on scientific evidence, the effects of antipyretics on fever patterns and child discomfort need to be clarified through double-blinded randomized clinical trials.

### Limitations and Future Work

In our study, fever was defined as a body temperature above 38°C regardless of measurement method or age [53]. Although in cases where rectal temperature measurements of 38°C or above, or 1°C or more above basal body temperature, may be defined as a fever [35,36], rectal temperature measurements are not a readily applicable method of measuring body temperature, and infrared tympanic thermometers, noncontact infrared-forehead thermometers, axillary thermometers, and oral thermometers are most commonly used in practice [54,55] with inconsistent results [56-58]. Body temperature may also vary depending on the age and biological factors of an individual. The dataset used in this study did not contain any information concerning the temperature measurement method or site, and it was difficult to analyze all relevant factors including age and biological factors in relation to temperature measurement. Therefore, we defined a conservative standard for fever as a body temperature greater than 38°C. This criterion was consistent with the body temperature at which the child was administered the antipyretic drug. A more accurate analysis would be possible using a definition of fever as a body temperature of 1°C above the basal body temperature or above 38°C, as well as obtaining the measurement method and site, and acquiring mandatory data on basal body temperature.

Data used here were obtained by app users directly entering their child’s body temperature. Since a uniform standard for measuring body temperatures was not applied (including measurement device and site), questions can be raised concerning the accuracy of the body temperature data. Furthermore, the app is dependent on the user entering the data correctly and consistently.

Despite these limitations, the five vaccines included in this study were more frequently recorded compared to the vaccines that we excluded. It is possible that postvaccination fever is actually more likely to occur due to these vaccines than others, and parents may also have believed that postvaccination fever in relation to these vaccines is more common than with others. Therefore, they may have been more likely to perform body temperature measurements. These limitations could be overcome through wearing thermometers and body temperature recording applications that continuously measure body temperatures before and after vaccination.

In practice, multiple vaccinations are recommended rather than single vaccinations, and more people are having multiple vaccinations, since there are no challenges in obtaining immunogenicity, no increase in side effects, and immunization schedules can be simplified [59]. However, multiple vaccinations may have an effect on postvaccination fever patterns depending on vaccine types. This study focused on single vaccinations; subsequent studies are underway concerning multiple vaccinations.

In our study, an analysis of the use of antipyretic drugs was only conducted after vaccination. However, the postvaccination fever pattern can vary depending on when an antipyretic drug is administered. More detailed research on postvaccination fever patterns is needed to determine when an antipyretic drug is to be administered.

Through creating a model that can predict future progress, based on the natural course of postvaccination fever (Figure 6), postvaccination temperature monitoring can provide useful information to parents, caregivers, and health care professionals, which is likely to reduce unnecessary tests and treatments, and consequently contribute to improvements in children’s health.

### Conclusion

Postvaccination fever has its own fever pattern depending on the type of vaccine administered. The pattern of postvaccination fever can be altered using antipyretic drugs, making the diagnosis of postvaccination fever difficult. This study showed that antipyretic drugs may prolong the duration of postvaccination fever, due to routine use or overuse. Postvaccination body temperature observation and comparison with postvaccination fever patterns indicated here may reduce the unnecessary use of antipyretics.

## Appendix

Multimedia Appendix 1 Screenshots of Fever Coach application translated into English.

Multimedia Appendix 2 Comparison of onset, offset, and duration times and maximum temperatures among vaccine types and effects of antipyretics on postvaccination fever response.

## Abbreviations

BCG: bacillus Calmette-Guérin
CI: confidence interval
DTaP: diphtheria, tetanus, and pertussis
HIB: Haemophilus influenzae type B
IQR: interquartile range
MMR: measles-mumps-rubella
PGHD: patient-generated health data
VIF: variance inflation factor
