# Correction: Postvaccination Fever Response Rates in Children Derived Using the Fever Coach Mobile App: A Retrospective Observational Study

**DOI:** 10.2196/18921

**Published:** 2020-05-07

**Authors:** Sang Hyun Ahn, Jooho Zhiang, Hyery Kim, Seyun Chang, Jaewon Shin, Myeongchan Kim, Yura Lee, Jae-Ho Lee, Yu Rang Park

**Affiliations:** 1 Korea Human Resource Development Institute for Health and Welfare Cheongju Republic of Korea; 2 Yonsei University College of Medicine Seoul Republic of Korea; 3 Department of Pediatrics University of Ulsan College of Medicine Asan Medical Center Children’s Hospital Seoul Republic of Korea; 4 Mobile Doctor Co, Ltd Seoul Republic of Korea; 5 Department of Biomedical Informatics Asan Medical Center Seoul Republic of Korea; 6 Department of Emergency Medicine Asan Medical Center University of Ulsan College of Medicine Seoul Republic of Korea; 7 Department of Biomedical Systems Informatics Yonsei University College of Medicine Seoul Republic of Korea

The authors of “Postvaccination Fever Response Rates in Children Derived Using the Fever Coach Mobile App: A Retrospective Observational Study” (JMIR Mhealth Uhealth 2019;7(4):e12223) identified an error in the Acknowledgements section.

In the first part of the Acknowledgements section, the following phrase:

This work was supported by Technology Innovation Program (10060085) funded by the Ministry of Trade, Industry, and Energy (MOTIE, KOREA)

Has been changed to:

This work was supported by Technology Innovation Program (20002289) funded by the Ministry of Trade, Industry, and Energy (MOTIE, KOREA)

The correction will appear in the online version of the paper on the JMIR website on May 7, together with the publication of this correction notice. Because this was made after submission to PubMed, PubMed Central, and other full-text repositories, the corrected article has also been resubmitted to those repositories.

