# Supplementary material for: Postvaccination Fever Response Rates in Children Derived Using the Fever Coach Mobile App: A Retrospective Observational Study
Source: JMIR Mhealth Uhealth. 2019 Apr 22;7(4):e12223. doi: 10.2196/12223 (PMC6658305; doi:10.2196/12223)
Supplement: Multimedia Appendix 1 [file mhealth_v7i4e12223_app1.pdf]

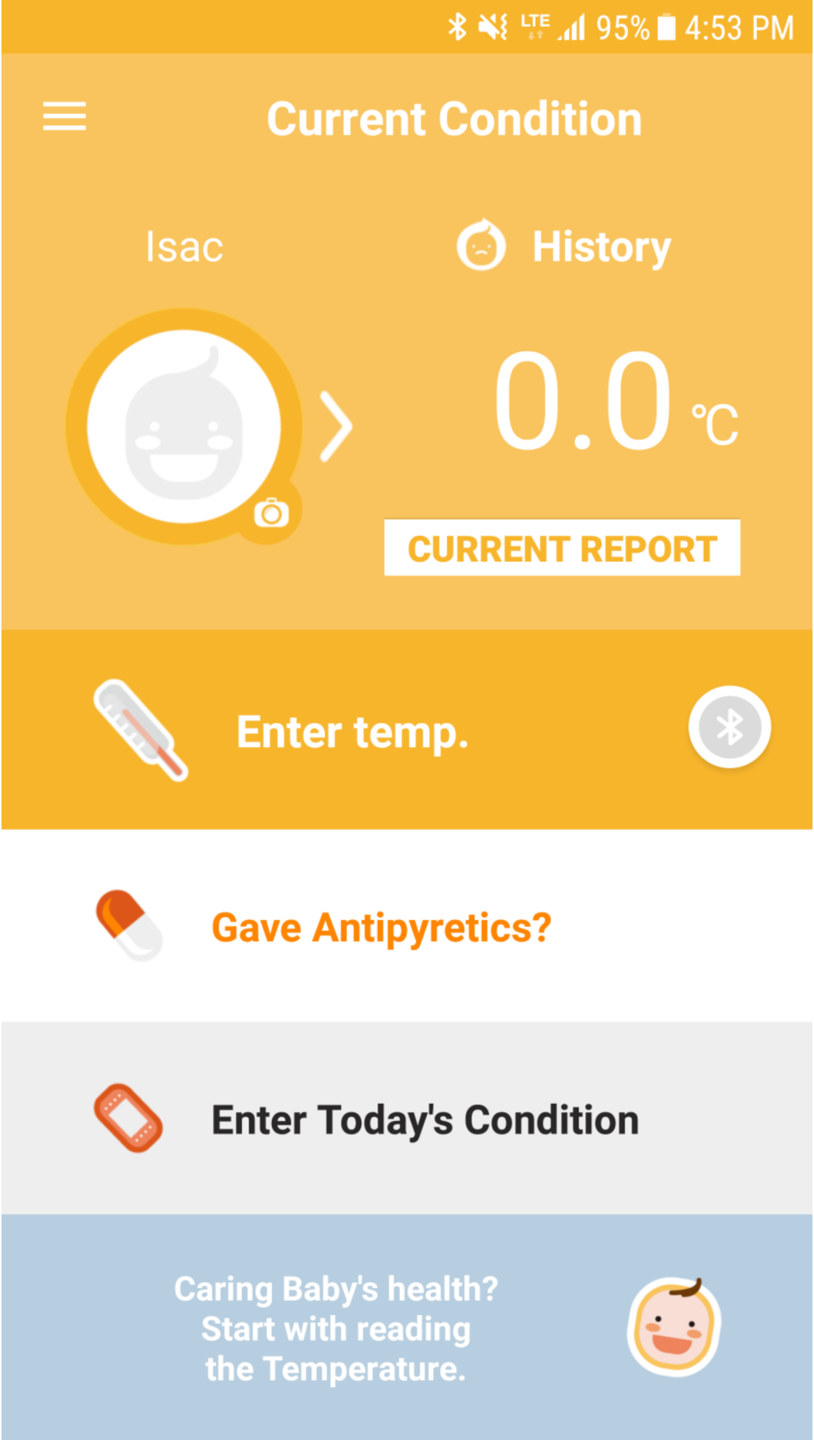
95% 4:53 PM
Current Condition
Isac
History
0.0 ℃
CURRENT REPORT
Enter temp.
Gave Antipyretics?
Enter Today's Condition
Caring Baby's health?
Start with reading
the Temperature.

95% 4:53 PM

Baby Information

Name	Isac
Birthday	2016-06-03
Gender	Male
Weight	12

Had convulsion

DELETE | OK

95% 4:54 PM

Enter the temperature

What is the temperature of child?

0.0 ℃

When did you measure the temperature?

2017-10-27

PM 04:54

Had convulsion

Have vaccinations within 24 hours, input here

Cancel

Ok

95% 4:54 PM

Enter the temperature

What is the temperature of child?

0.0

Enter the temperature

36		
37		
38	. 0	℃
39	1	
40	2	

CANCEL OK

Had convulsion

Have vaccinations within 24 hours, input here

Cancel Ok

4:54 PM 95%

Enter the temperature

What is the temperature of child?

39.0 ℃

When did you measure the temperature?

2017-10-27

PM 04:54

Had convulsion

Have vaccinations within 24 hours, input here

Cancel

Ok

LTE 95% 4:54 PM

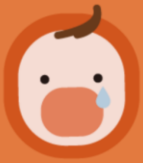

Fever

38.0℃

The child has a fever.

Measure again after 1 hours.

Consider giving antipyretics if the child is suffering from the fever or the body starts to sag.

Use acetaminophen type or ibuprofen type. A proper dose is 5.0cc ~ 6.0cc.

Antipyretics ▼

Make sure to give the child a plenty of water.

Calculate the water intake

If you are not giving the child antipyretic, wipe off the child's face, neck and chest with lukewarm water.

[illegible]

Ok

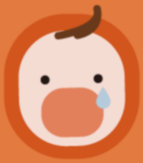

Fever

38.0℃

The child has a fever.

Measure again after 1 hours.

Consider giving antipyretics if the child is suffering from the fever or the body starts to sag.

Use acetaminophen type or ibuprofen type. A proper dose is 5.0cc ~ 6.0cc.

Antipyretics ▲

* Acetaminophen Type: Tylenol, Setopen, Champ, Tanophen, etc.
* Ibuprofen Type: Brufen, Carol, Green Fan, Ibufen Daewoong, etc.
* Dose for pills or suppositories
- 1 tablet of Tylenol 80mg is equivalent to 2.5cc of syrup.
- 1 tablet of Suspen Suppositories 125mg is equivalent to 4cc of syrup.

Ok

Consider giving antipyretics if the child is suffering from the fever or the body starts to sag.

Use acetaminophen type or ibuprofen type. A proper dose is 5.0cc ~ 6.0cc.

Antipyretics ▲

* Acetaminophen Type: Tylenol, Setopen, Champ, Tanophen, etc.
* Ibuprofen Type: Brufen, Carol, Green Fan, Ibufen Daewoong, etc.
* Dose for pills or suppositories
- 1 tablet of Tylenol 80mg is equivalent to 2.5cc of syrup.
- 1 tablet of Suspen Suppositories 125mg is equivalent to 4cc of syrup.

Make sure to give the child a plenty of water.

Calculate the water intake

If you are not giving the child antipyretic, wipe off the child's face, neck and chest with lukewarm water.

Instructions for lukewarm water massage ▼

Ok

95% 4:54 PM

[illegible]

Antipyretics ▲

* Acetaminophen Type: Tylenol, Setopen, Champ, Tanophen, etc.
* Ibuprofen Type: Brufen, Carol, Green Fan, Ibufen Daewoong, etc.
* Dose for pills or suppositories
- 1 tablet of Tylenol 80mg is equivalent to 2.5cc of syrup.
- 1 tablet of Suspen Suppositories 125mg is equivalent to 4cc of syrup.

Make sure to give the child a plenty of water.

Calculate the water intake

If you are not giving the child antipyretic, wipe off the child's face, neck and chest with lukewarm water.

Instructions for lukewarm water massage ▲

Wipe off face, neck, chest and armpits of the child with lukewarm water of 32~35℃ without squeezing it. Do not wipe off arms or leg, it could contract the peripheral nerves.

Ok

94% 4:55 PM

Enter the dose.

Syrup | Powder | Pill | Suppository

Tylenol, Setopen, Champ, etc.
Acetaminophen type

✓ Brufen, Carol, etc.
Ibuprofen type

Mexibufen, etc.
Dexibuprofen type

Antipyretic's name (optional)

Advil0

Given dose

11

Given at

2017-10-27 | PM 04:54

Enter the dose.

Mexibufen, etc.
Dexibuprofen type

Antipyretic's name (optional)

Advil0

Given dose

11

Given at

2017-10-27 PM 04:54

Tab if the current weight isn't 12kg

※ Tab into the dosage given to child.

Cancel Ok

94% 4:56 PM

Today's Records

Visited hospital

2017-10-27 | PM 04:56

Select a disease

Memo

Leave the doctor's instructions or diagnosis details.

Cancel save

94% 4:56 PM

Today's Records

Got vaccination

2017-10-27

PM 04:56

Select vaccinations

Memo

You can edit/delete on History > Timeline

Cancel

save

fever coach

- Home
- Baby info
- History
- Memo
- Measurement tips
- Report
- Settings

MOBILE DOCTOR

4:56 PM 94%

History

Graph

Timeline

2017-10-27

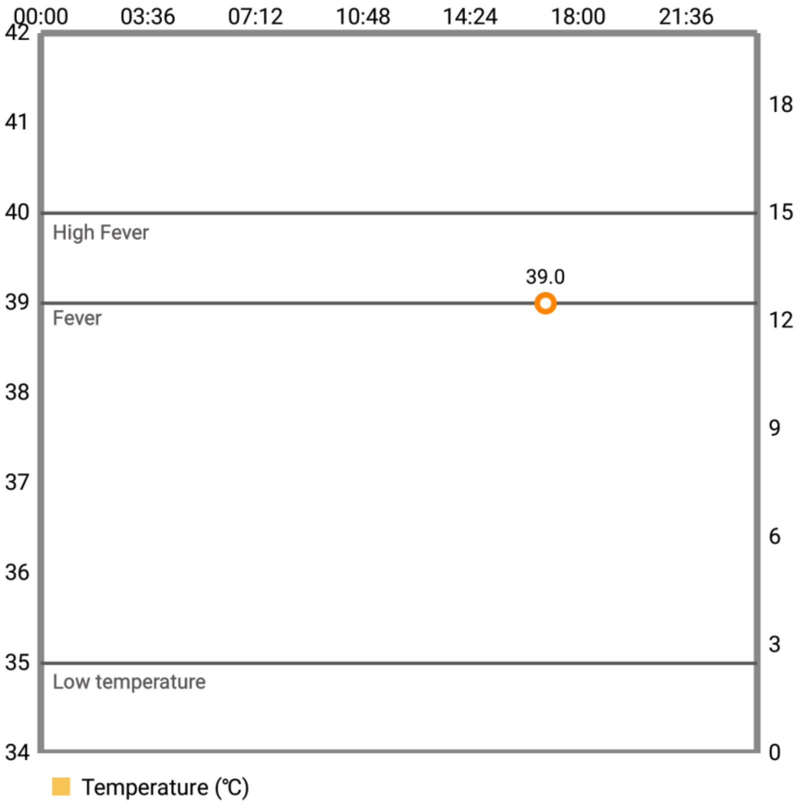

92% 5:03 PM

Baby Information

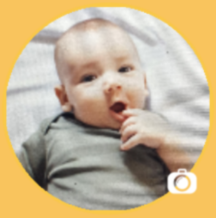

Name	Isac
Birthday	2016-06-03
Gender	Male
Weight	12

Had convulsion

DELETE OK

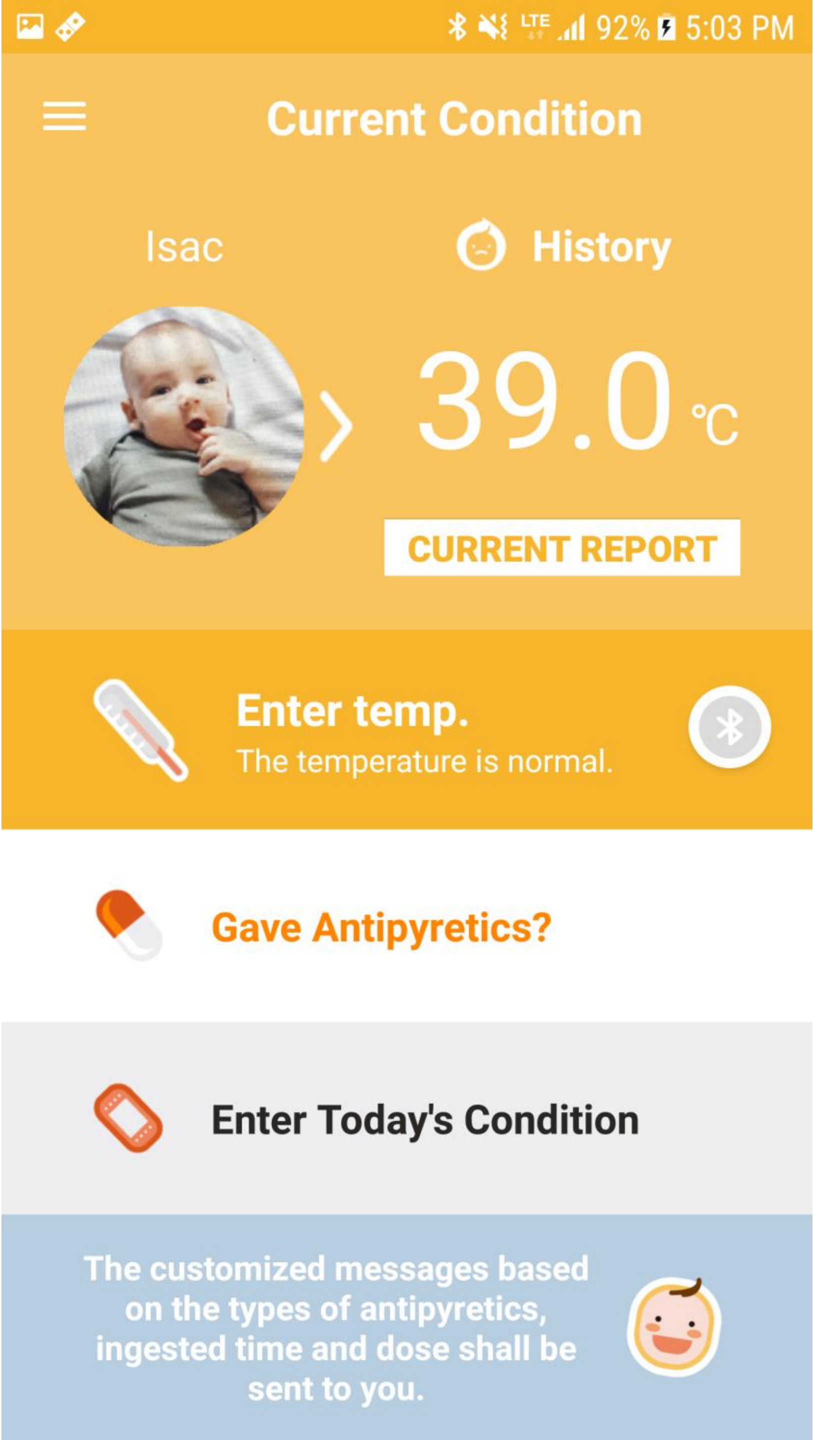
92% 5:03 PM
Current Condition
Isac
History
39.0 ℃
CURRENT REPORT
Enter temp.
The temperature is normal.
Gave Antipyretics?
Enter Today's Condition
The customized messages based on the types of antipyretics, ingested time and dose shall be sent to you.
