# Supplementary material for: Postvaccination Fever Response Rates in Children Derived Using the Fever Coach Mobile App: A Retrospective Observational Study
Source: JMIR Mhealth Uhealth. 2019 Apr 22;7(4):e12223. doi: 10.2196/12223 (PMC6658305; doi:10.2196/12223)
Supplement: Multimedia Appendix 2 [file mhealth_v7i4e12223_app2.docx]

**Multimedia Appendix 2 Comparison of onset, offset, duration time and max temperature between vaccine records with and without antipyretics**

| Vaccine type | onset time (mean ± SD) | | p-value | offset time (mean ± SD) | | p-value | duration time (mean ± SD) | | p-value | Max temperature (mean ± SD) | | p-value |
| --- | --- | --- | --- | --- | --- | --- | --- | --- | --- | --- | --- | --- |
|  | with | without |  | with | without |  | with | without |  | with | without |  |
| Pneumococcus | 9.0±8.3 | 9.7±8.0 | .04 | 20.4±12.5 | 12.5±8.6 | <.001 | 11.5±11.5 | 2.8±4.2 | <.001 | 38.7±0.6 | 38.3±0.4 | <.001 |
| DTaP | 12.8±10.9 | 14.1±11.9 | .35 | 26.0±16.6 | 16.6±11.7 | <.001 | 13.2±11.2 | 2.5±3.5 | <.001 | 38.9±0.7 | 38.3±0.4 | <.001 |
| Hepatitis A | 13.7±11.4 | 15.3±13.4 | .56 | 31.1±12.1 | 20.1±12.8 | <.001 | 17.4±11.8 | 4.9±7.5 | <.001 | 39.0±0.6 | 38.4±0.4 | <.001 |
| Influenza | 13.1±11.1 | 12.3±12.9 | .54 | 28.8±12.7 | 18.4±13.7 | <.001 | 15.7±12.2 | 6.1±8.3 | <.001 | 39.0±0.6 | 38.5±0.8 | <.001 |
| Japanese encephalitis | 10.9±10.0 | 9.4±9.9 | .15 | 24.7±13.5 | 14.0±10.7 | <.001 | 13.8±11.8 | 4.6±6.2 | <.001 | 38.9±0.6 | 38.4±0.5 | <.001 |
| All | 10.9±9.9 | 10.3±0.3 | .12 | 24.3±13.4 | 13.6±9.9 | <.001 | 13.4±11.9 | 3.2±5.0 | <.001 | 38.8±0.6 | 38.3±0.5 | <.001 |
